# Supplementary material for: A calibratable sensory neuron based on epitaxial VO2 for spike-based neuromorphic multisensory system
Source: Nat Commun. 2022 Jul 8;13:3973. doi: 10.1038/s41467-022-31747-w (PMC9270461; doi:10.1038/s41467-022-31747-w)
Supplement: Supplementary file 1 — Supplementary Information [file 41467_2022_31747_MOESM1_ESM.pdf]

Supplementary Information

A Calibratable Sensory Neuron Based on Epitaxial VO_2 for Spike-based Neuromorphic Multisensory System

Yuan et al.

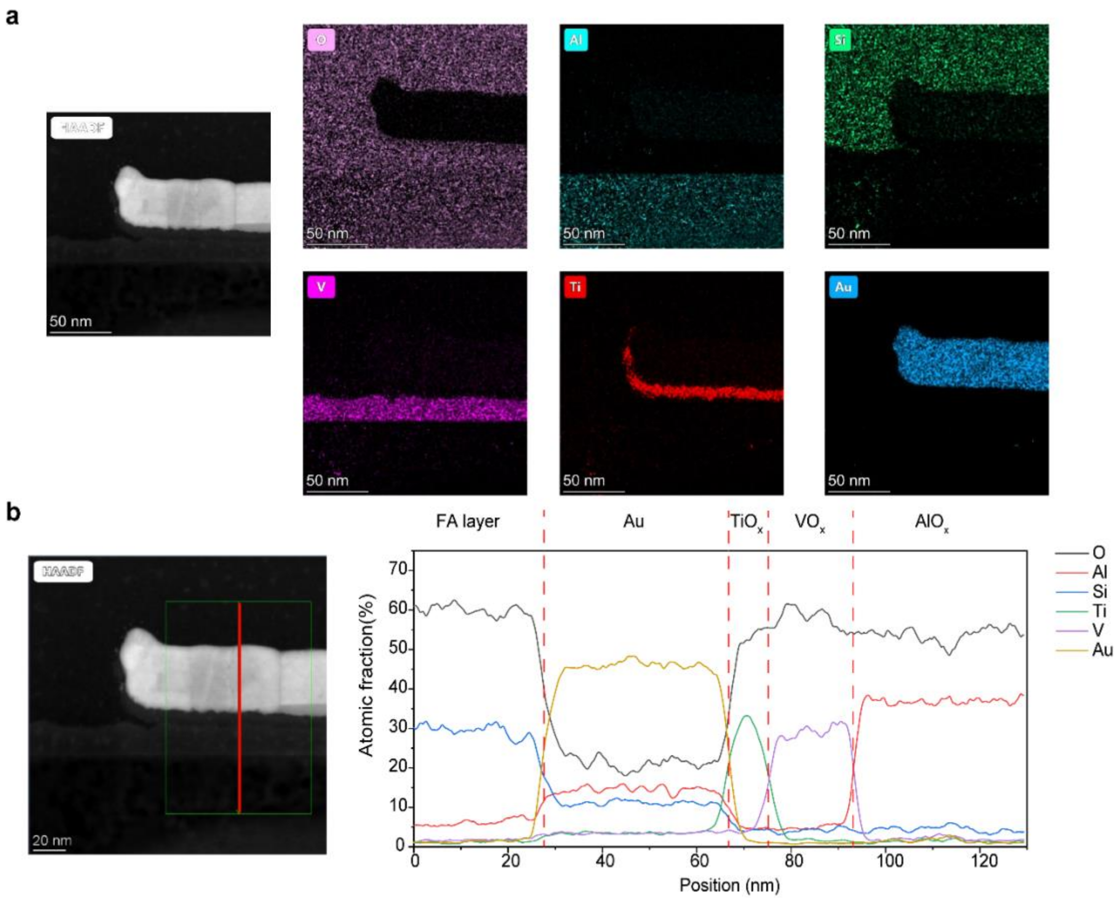

Supplementary Figure 1. Microstructural and compositional characterization of epitaxial VO_2 device. **(a)** Cross-sectional STEM image and corresponding EDS mapping of Al, O, Au, Ti, Si and V elements in the device. **(b)** EDS elemental line profile in the region of the device shown by the STEM image.

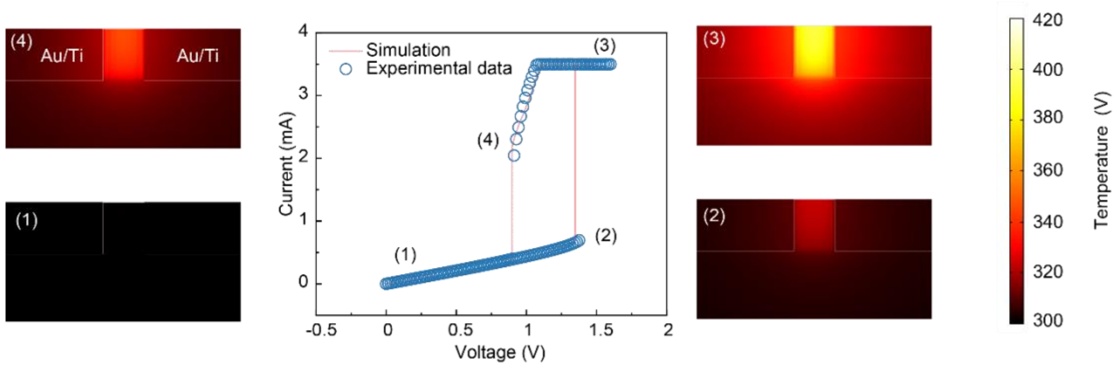

Supplementary Figure 2. Simulation results. The experimental *I-V* curve can be well fitted using our model. The insets show spatial heat distribution in different stages of the phase transition.

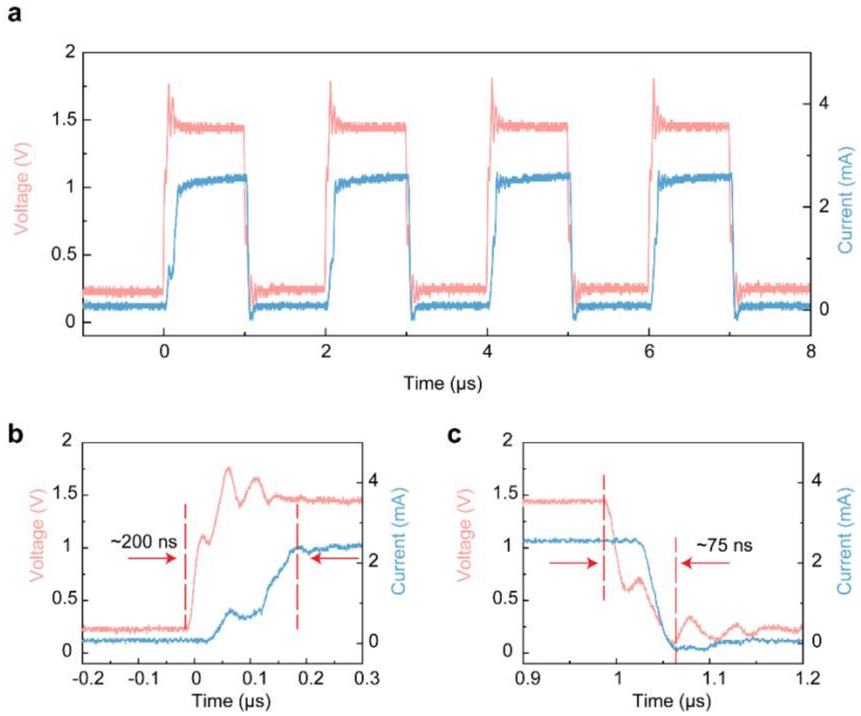

Supplementary Figure 3. Transient switching response of the VO_2 threshold switching device. **(a)** Current waveform (blue curve) of the VO_2 device upon application of the voltage waveform (pink curve). **(b)** The switching speed is < 200 ns from off- to on-state. **(c)** The switching speed is <75 ns from on- to off-state.

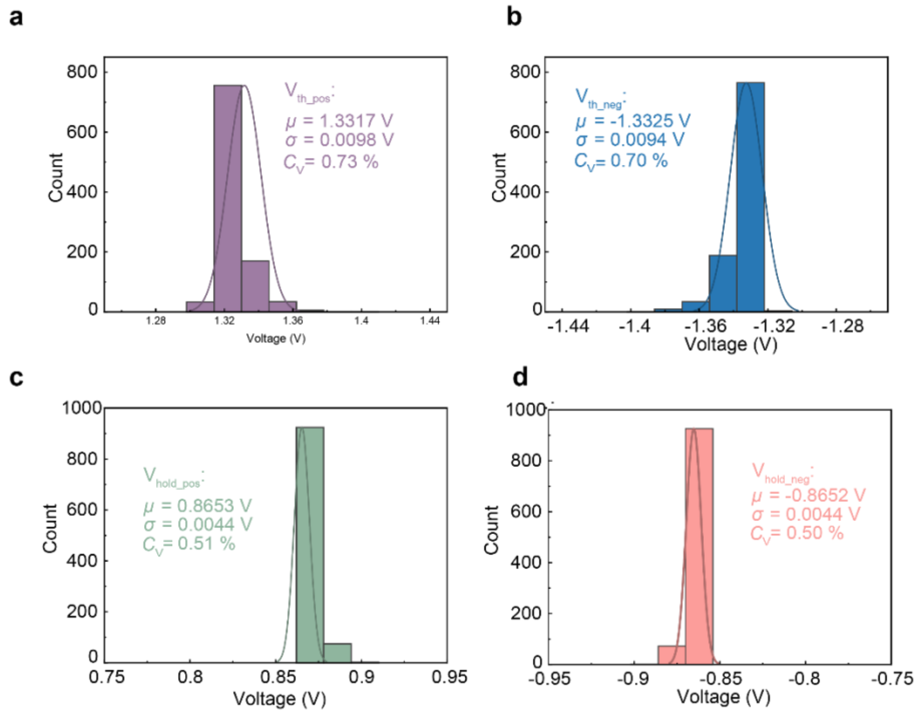

Supplementary Figure 4. The statistical analysis of variability in positive and negative threshold/holding voltages in 1000 cycles. (a) The variability of V_{th_pos}. **(b)** The variability of V_{th_neg}. **(c)** The variability of V_{hold_pos}, **(d)** The variability of V_{hold_neg}.

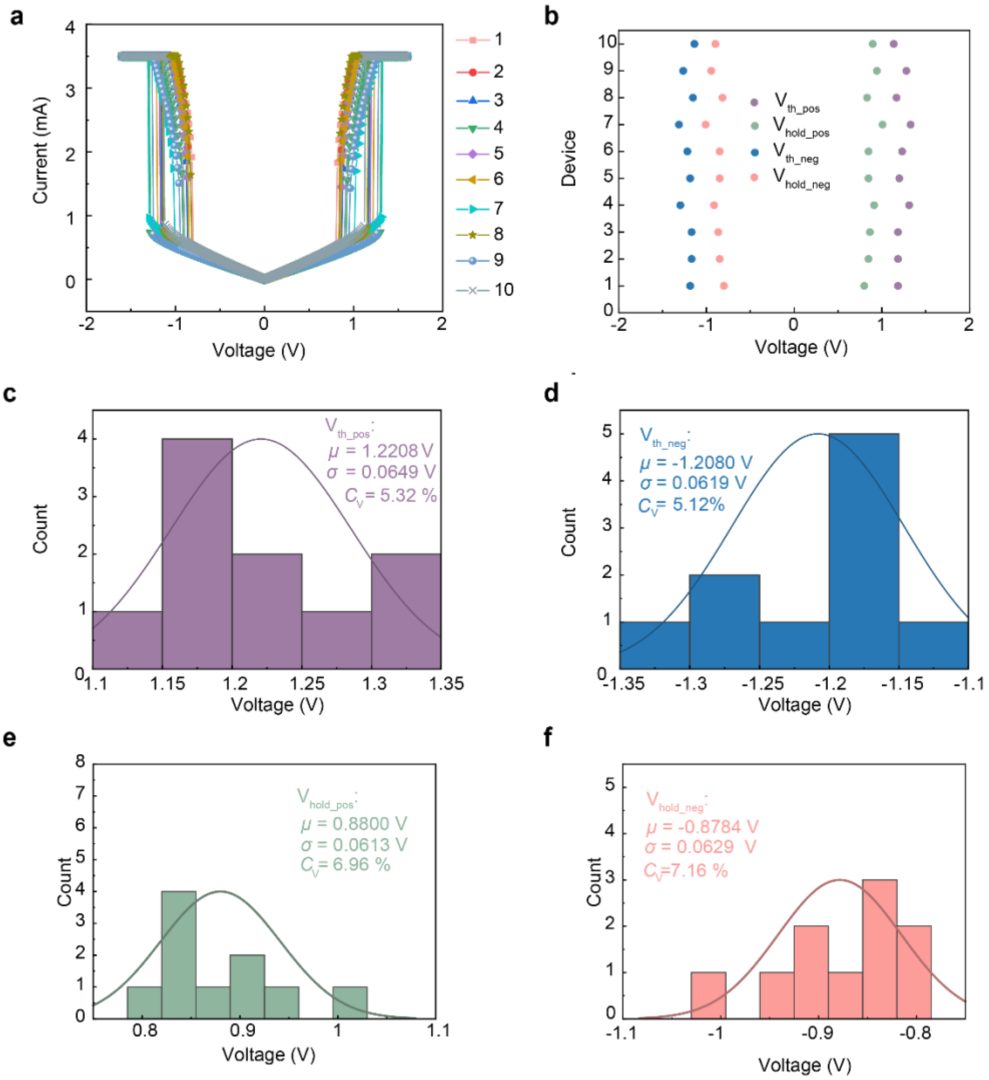

Supplementary Figure 5. *I-V* characteristics and statistical analysis of variability in threshold/holding voltages of 10 epitaxial VO_2 memristors. **(a)** *I-V* characteristics of the device measured in 10 different epitaxial VO_2 devices. **(b)** The V_{th_pos}, V_{hold_pos}, V_{th_neg} and V_{hold_neg} of 10 epitaxial VO_2 devices. **(c)-(f)** The statistical analysis of variability of V_{th_pos}, V_{th_neg}, V_{hold_pos} and V_{hold_neg} in 10 epitaxial VO_2 devices.

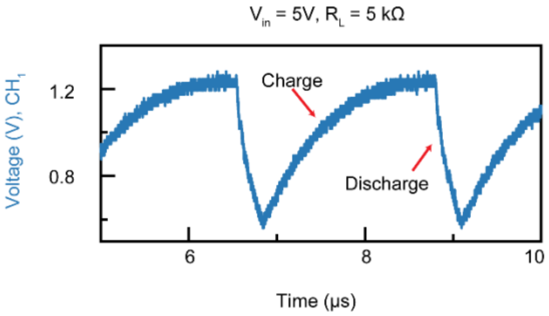

Supplementary Figure 6. A single oscillation characteristic of the spiking neuron. A fixed voltage of 5 V is applied, while a fixed series resistance is set at 5 kΩ. The process of charging and discharging can be observed clearly.

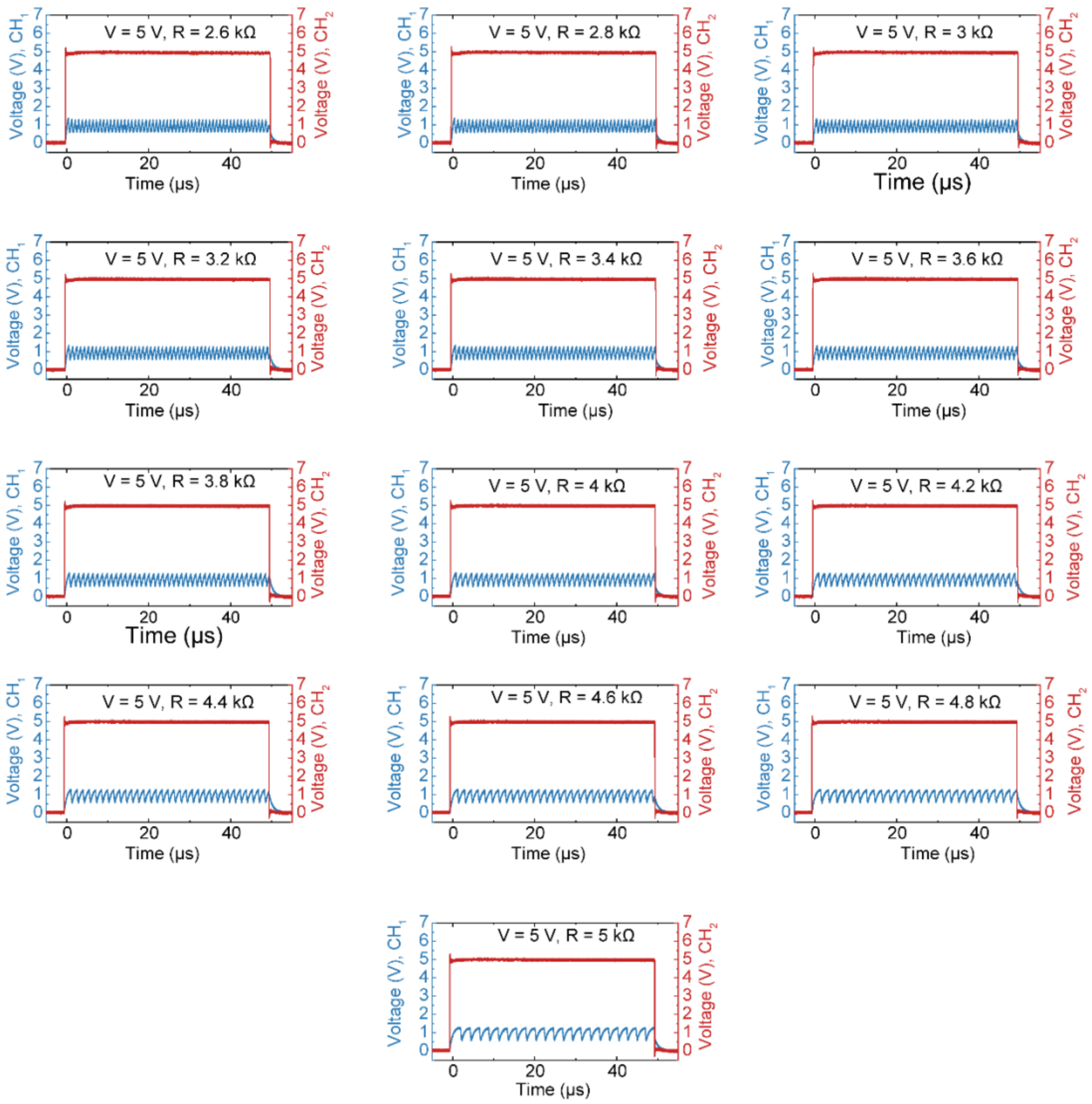

Supplementary Figure 7. The output results of spiking neuron using different series resistance. This figure shows the additional data under different series resistance R_L. As the series resistance increases, the spiking frequency gradually decreases. This is because as the series resistance increases, the input current gradually decreases and the charge accumulation becomes slower.

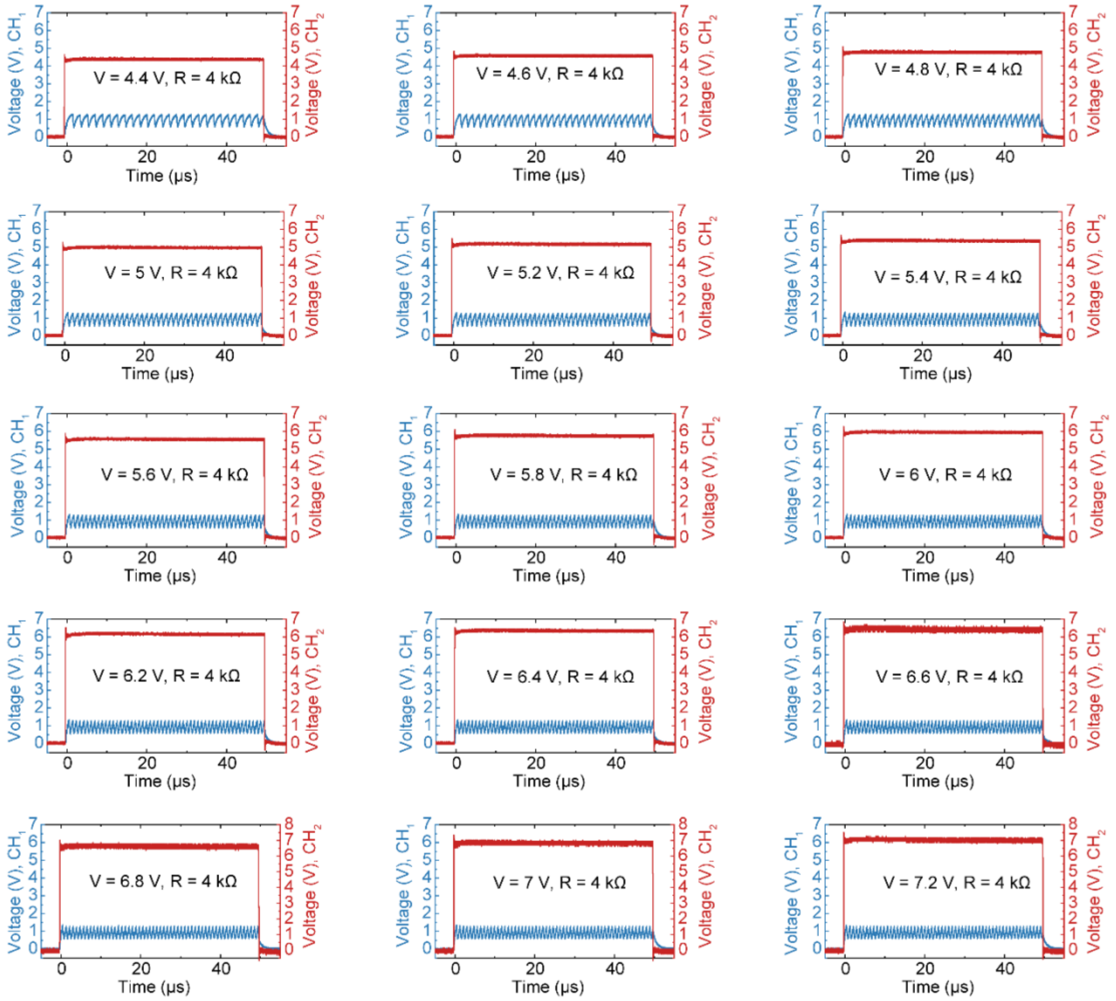

Supplementary Figure 8 The output results of spiking neuron under different applied voltage. This figure shows the additional data under different input voltages. As the applied voltage increases, the spiking frequency gradually increases.

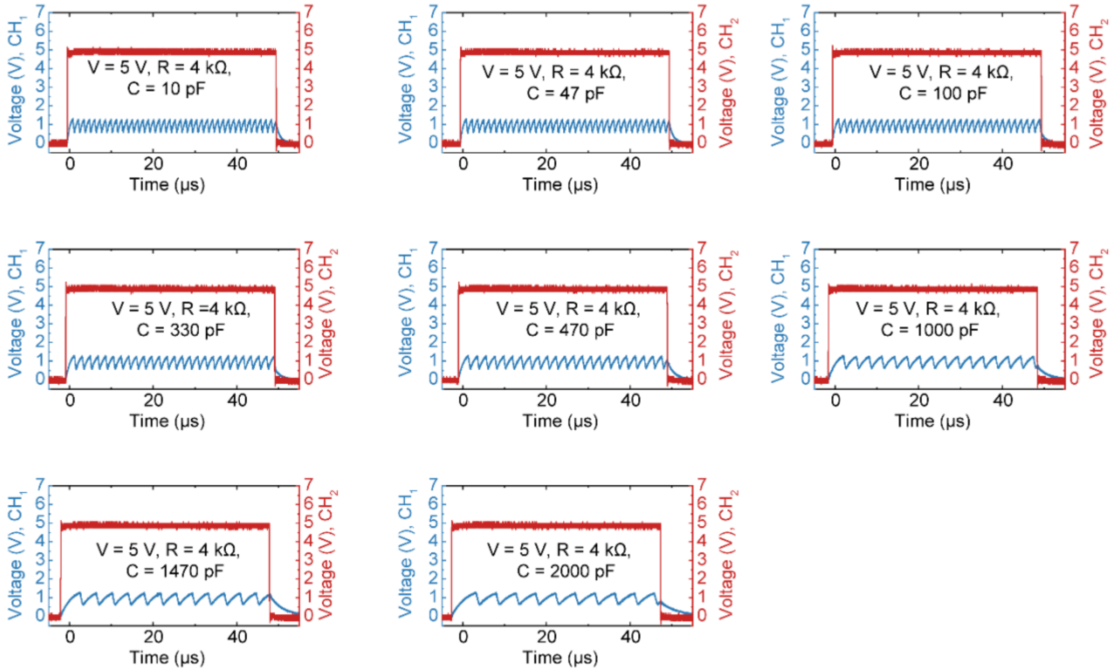

Supplementary Figure 9. The output results of spiking neuron under different parallel capacitors. This figure shows the additional data under different parallel capacitors that are not presented in Fig. 3. As the parallel capacitance increases, the firing frequency gradually decreases, this is because larger capacitance makes the integration process slower.

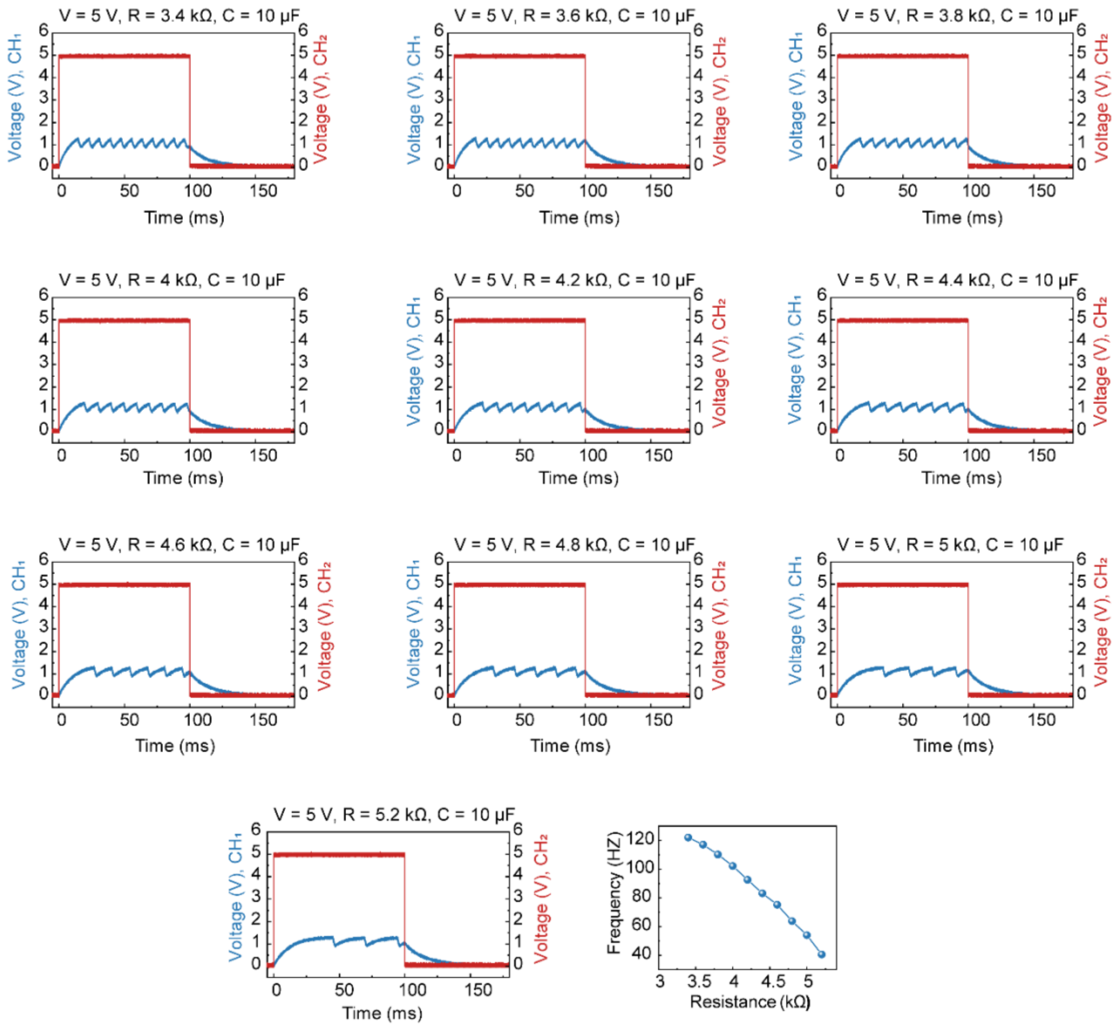

Supplementary Figure 10. The output results of spiking neuron under different series resistance with a 10 μF parallel capacitor. This figure shows the output results of the spiking neuron using different series resistance with a 10 μF parallel capacitor. A spiking frequency under 150 Hz is obtained which is similar to the human nervous system.

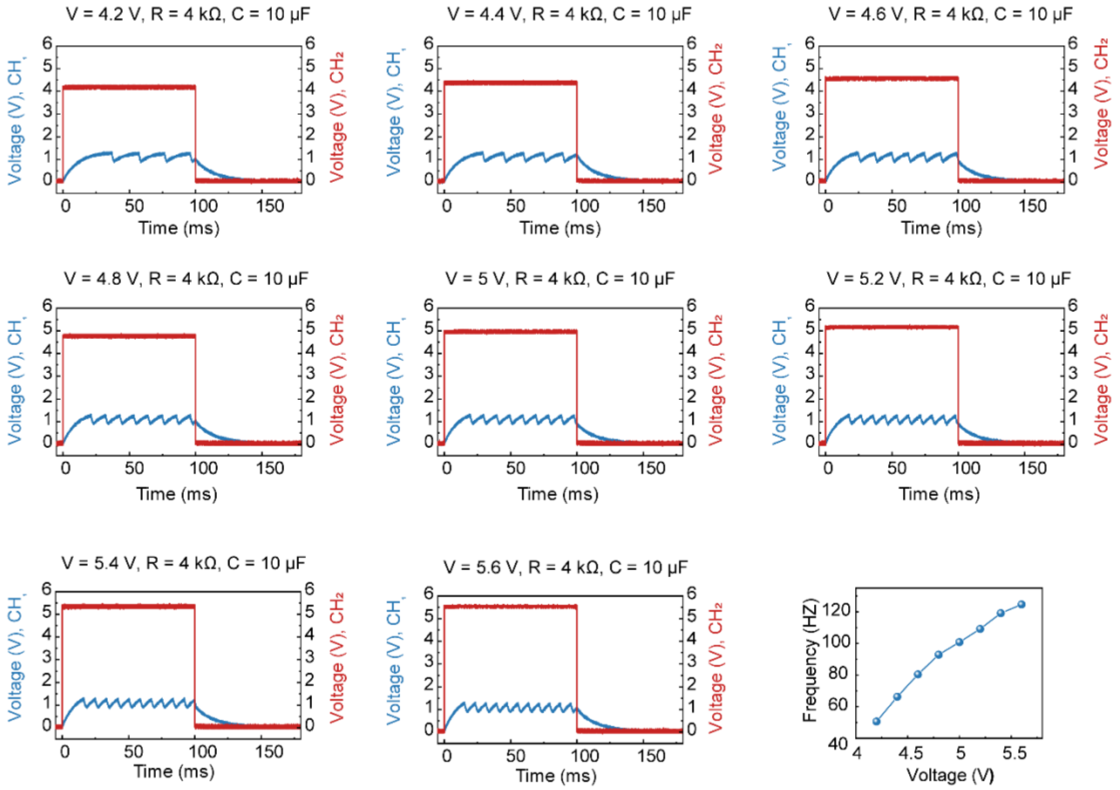

Supplementary Figure 11. The output results of spiking neuron under different applied voltages with a 10 μF parallel capacitor. This figure shows the output results of the spiking neuron using different applied voltages with a 10 μF parallel capacitor. A spiking frequency under 150 Hz is obtained which is similar to the human nervous system.

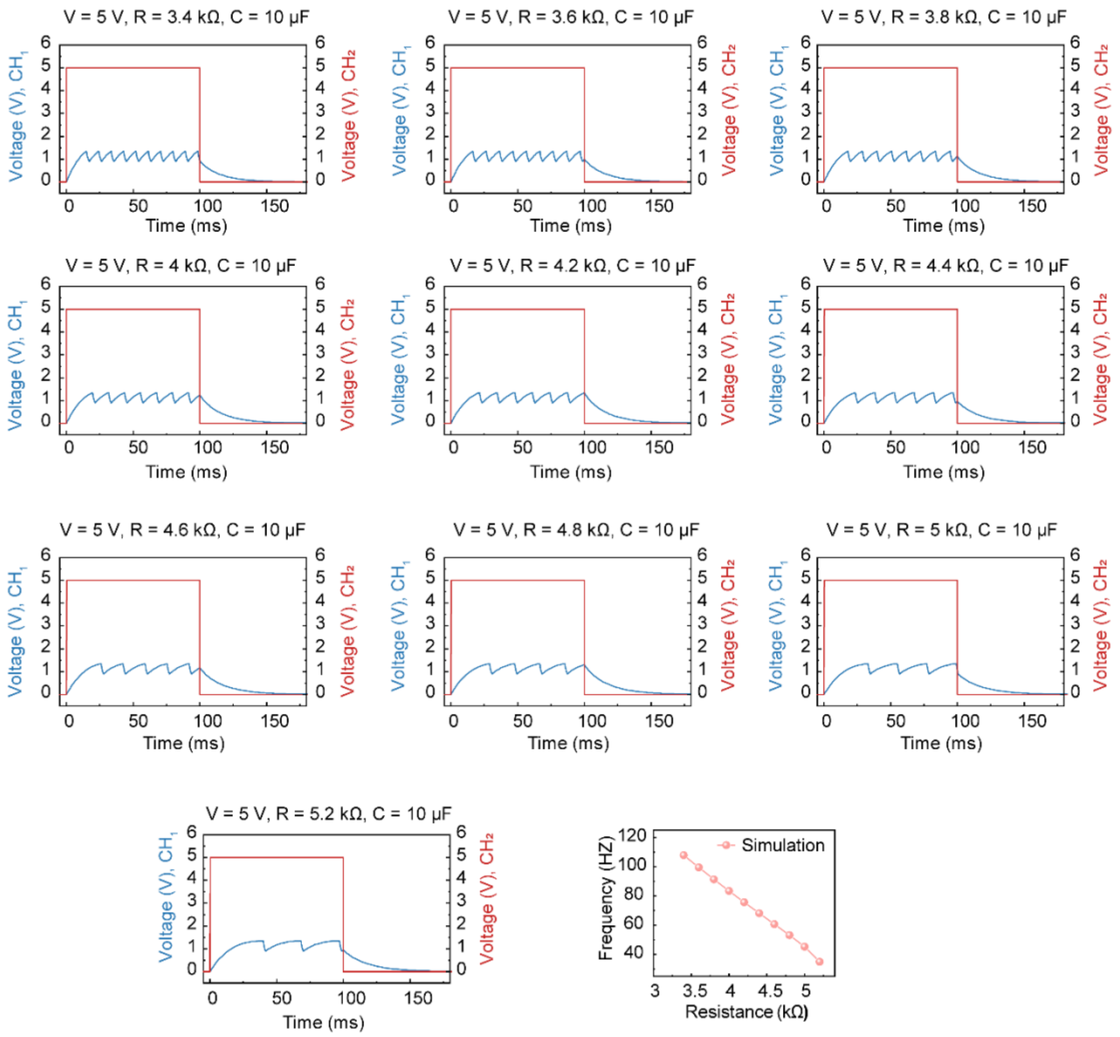

Supplementary Figure 12. Simulation results of spiking neuron under different series resistance with a 10 μF parallel capacitor. This figure shows the simulation results of the spiking neuron under different series resistance with a 10 μF parallel capacitor. Results from simulations and experiments are highly congruent.

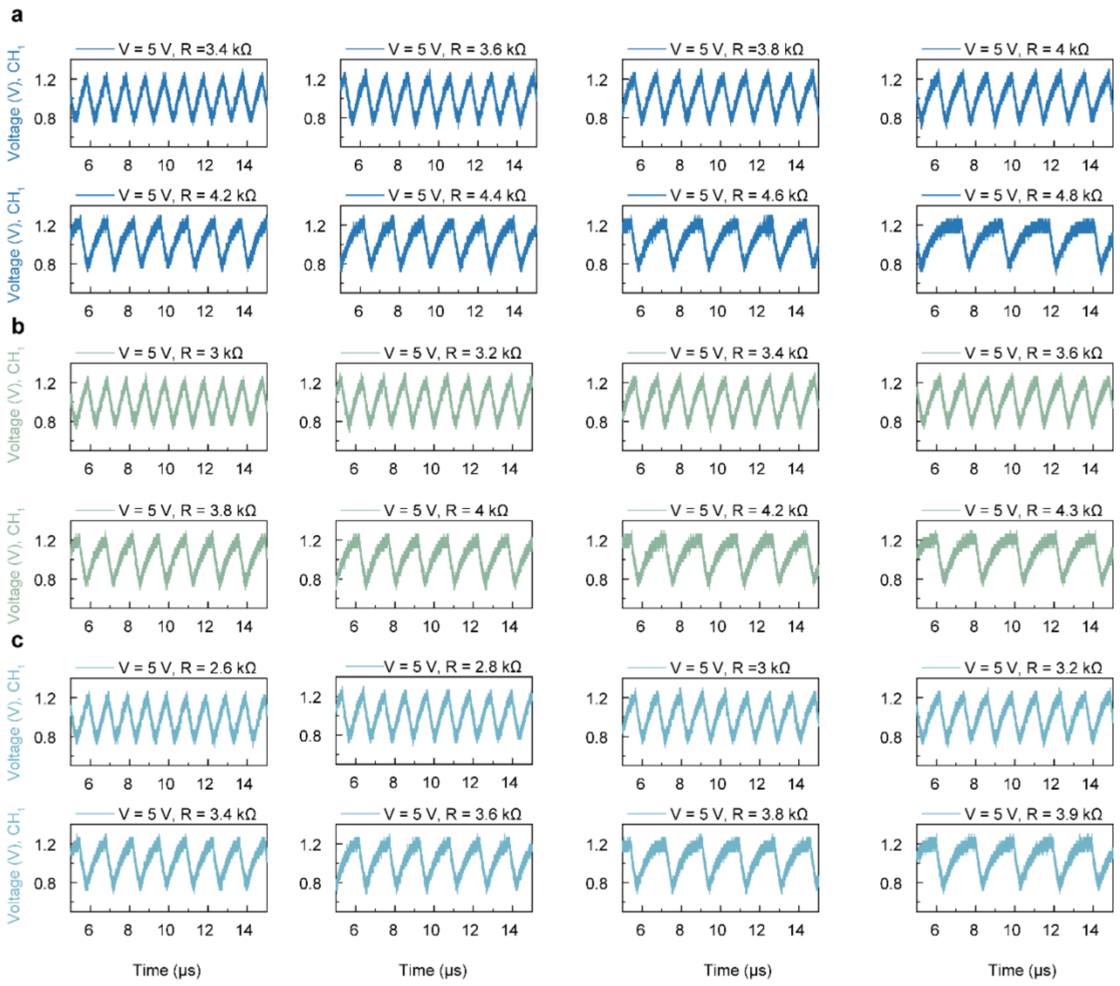

Supplementary Figure 13. The relationship between neuron spiking frequency and series resistance R_L under different calibration resistances R_c. This figure shows the additional data under different calibration resistances R_c that not presented in Fig. 3k. **(a)** The relationship between neuron spiking frequency and series resistance R_L when R_c is set as 0. **(b),** The relationship between neuron spiking frequency and series resistance R_L when R_c is set as 0.4 kΩ. **(c),** The relationship between neuron spiking frequency and series resistance R_L when Rc is set as 0.8 kΩ. This figure demonstrates that the relationship between spiking frequency and R_L can be controlled by the calibration resistor R_c.

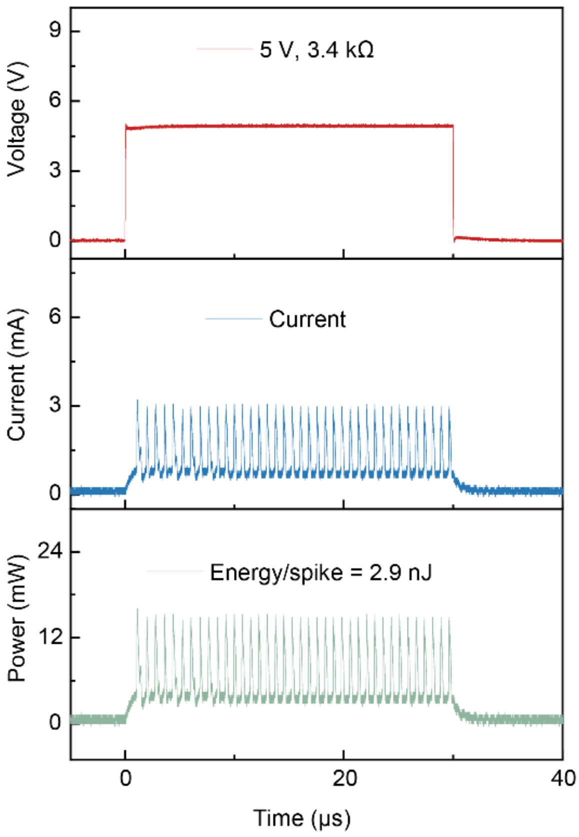

Supplementary Figure 14. The energy consumption of spiking neuron. Transient power is calculated by multiplication of input voltage by output current, and energy consumption is calculated by dividing the total consumption by the spike number.

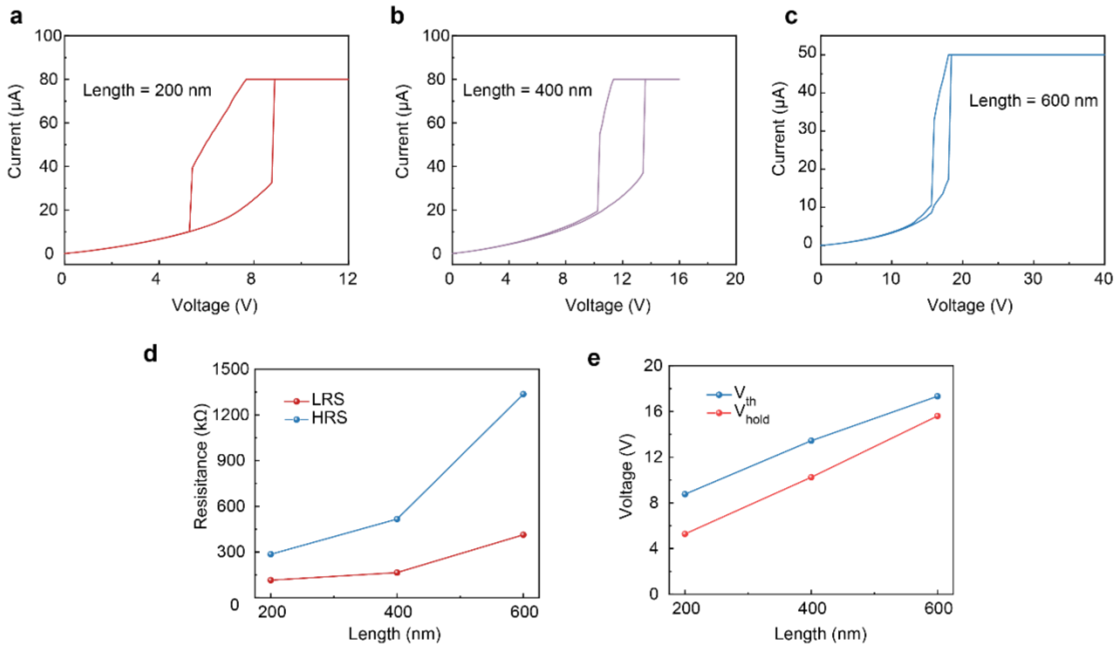

Supplementary Figure 15. The characteristics of the optimized VO_2 memristor. (a-c) The *I*-*V* characteristics of devices with different channel lengths. **(d)** Resistance of devices with different channel lengths. **(e)** V_{th} and V_{hold} for devices with different channel lengths.

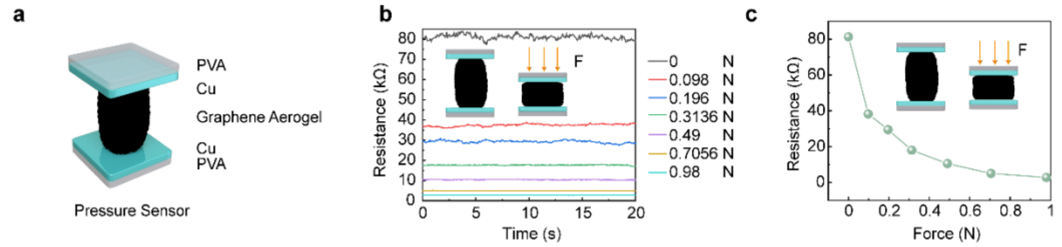

Supplementary Figure 16. The pressure sensor based on graphene aerogel. (a) Schematic diagram of graphene aerogel pressure sensor. By sandwiching a layer of graphene aerogel between two thin copper sheets, and then attaching a PVA protective film to the outside of the copper sheet, the sensor is very easy to prepare. (**b)** Response of graphene aerogel pressure sensor under different pressures. (**c)** The relationship between the pressure and the resistance of the graphene pressure sensor.

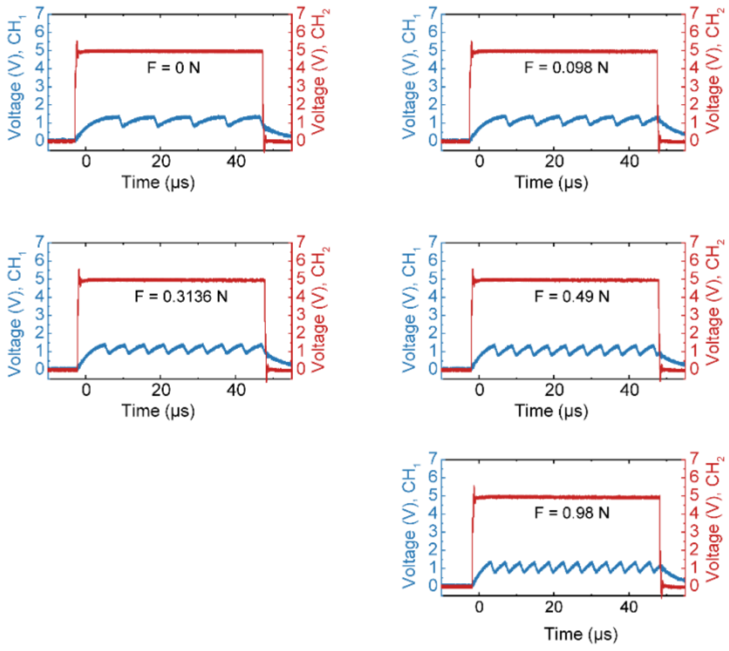

Supplementary Figure 17. Spiking frequency response under different pressure. As the pressure increases, the frequency of spiking increases.

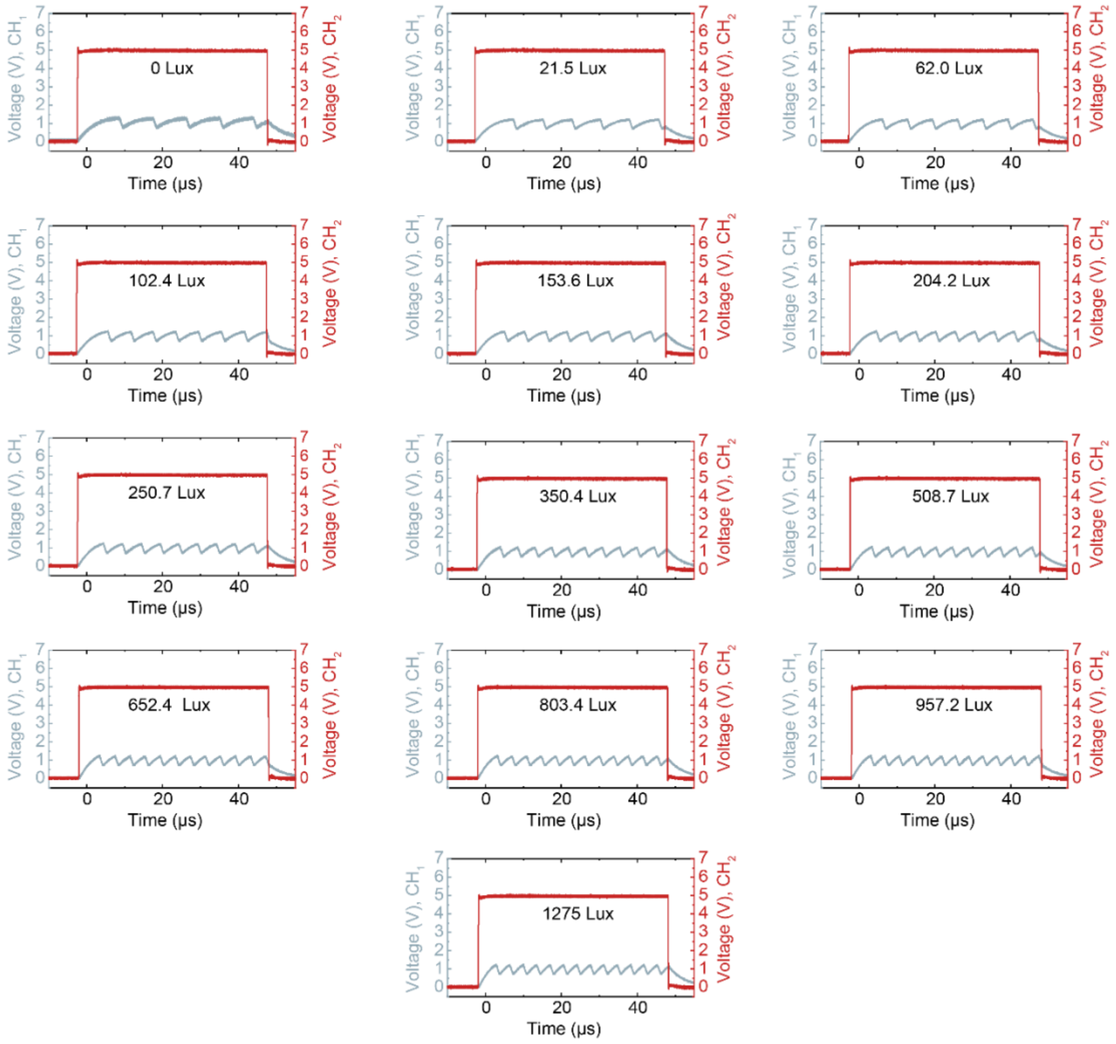

Supplementary Figure 18. Spiking frequency response under different illuminance. As the illuminance increases, the frequency of spiking increases.

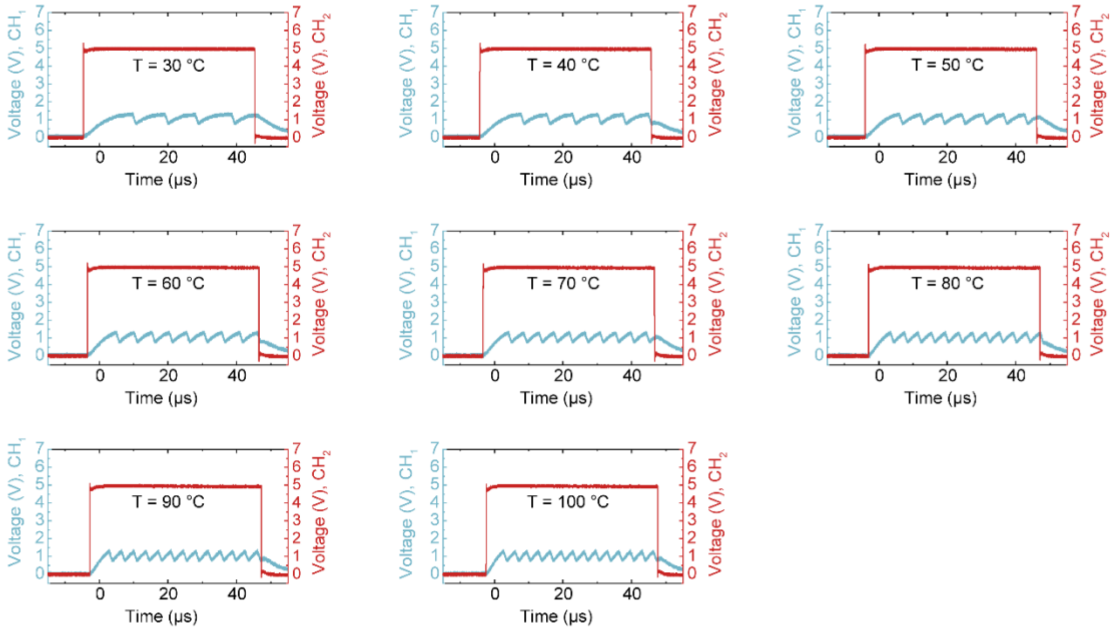

Supplementary Figure 19. Spiking frequency response under different temperatures.

As the temperature increases, the frequency of spiking increases.

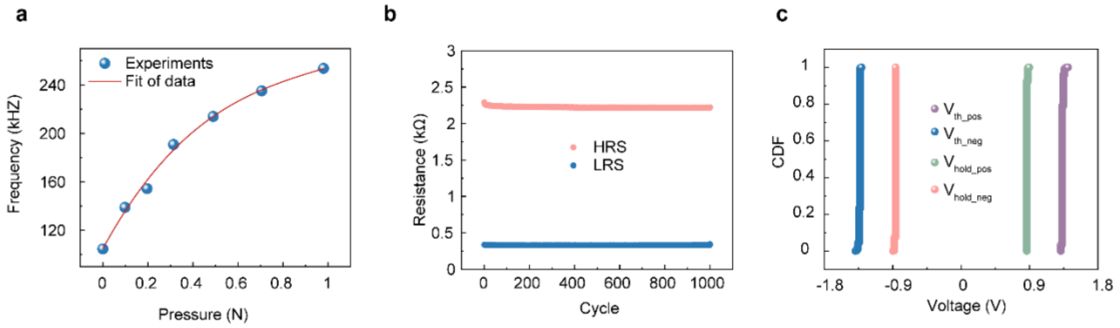

Supplementary Figure 20. Simulation of the spike-based neuromorphic perception and computing system for pressure image recognition. (a) The effect of pressure on spiking firing frequency of artificial spiking tactile sensory neuron. The blue point is the experimental data, the red curve is the fitted curve. **(b)** Distributions of high and low resistance states of the epitaxial VO_2 device in 1000 repeated cycles. In our simulation, the high resistance of the device is set to 2.2 kΩ, and the series resistance R_L is set to 4 kΩ. **(c),** Cumulative plots of V_{th_pos}, V_{hold_pos}, V_{th_neg}, and V_{hold_neg}. V_{th} is set to 1.4 V and V_{hold} to 0.85 V in our simulation.

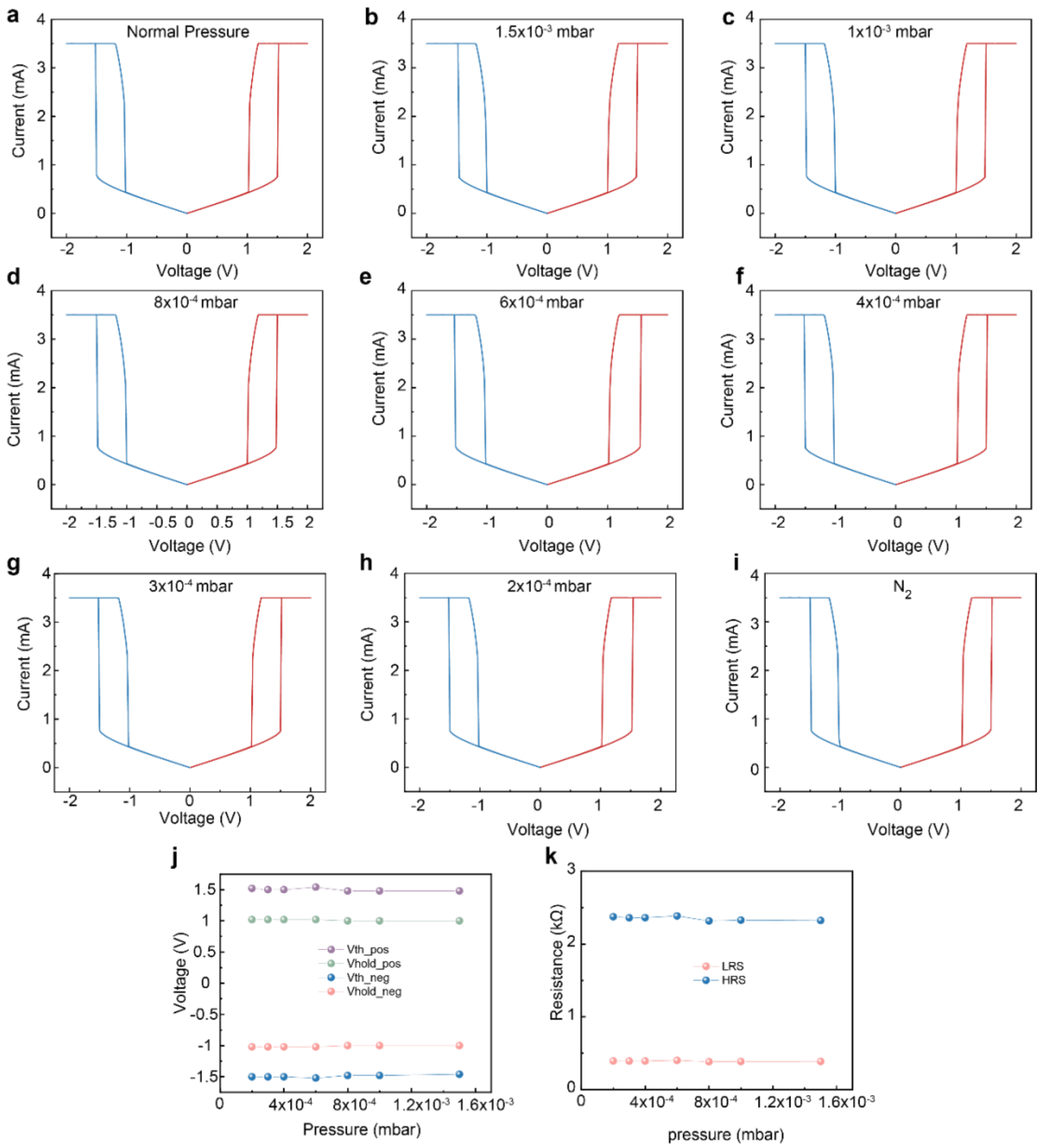

Supplementary Figure 21. Characteristics of the VO_2 memristor under different environment. (a) The *I-V* characteristics of devices in normal atmospheric pressure. **(b)-(i)** The *I-V* characteristics of devices in different atmospheric pressure which is changed from 1.5×10^{-3} mbar to 2×10^{-4} and in N_2 environment. The *I-V* characteristics of devices are stable. **(j)** The V_{th_pos}, V_{th_neg}, V_{hold_pos} and V_{hold_neg} at different atmospheric pressures. **(k)** The resistive states of devices at different atmospheric pressures.

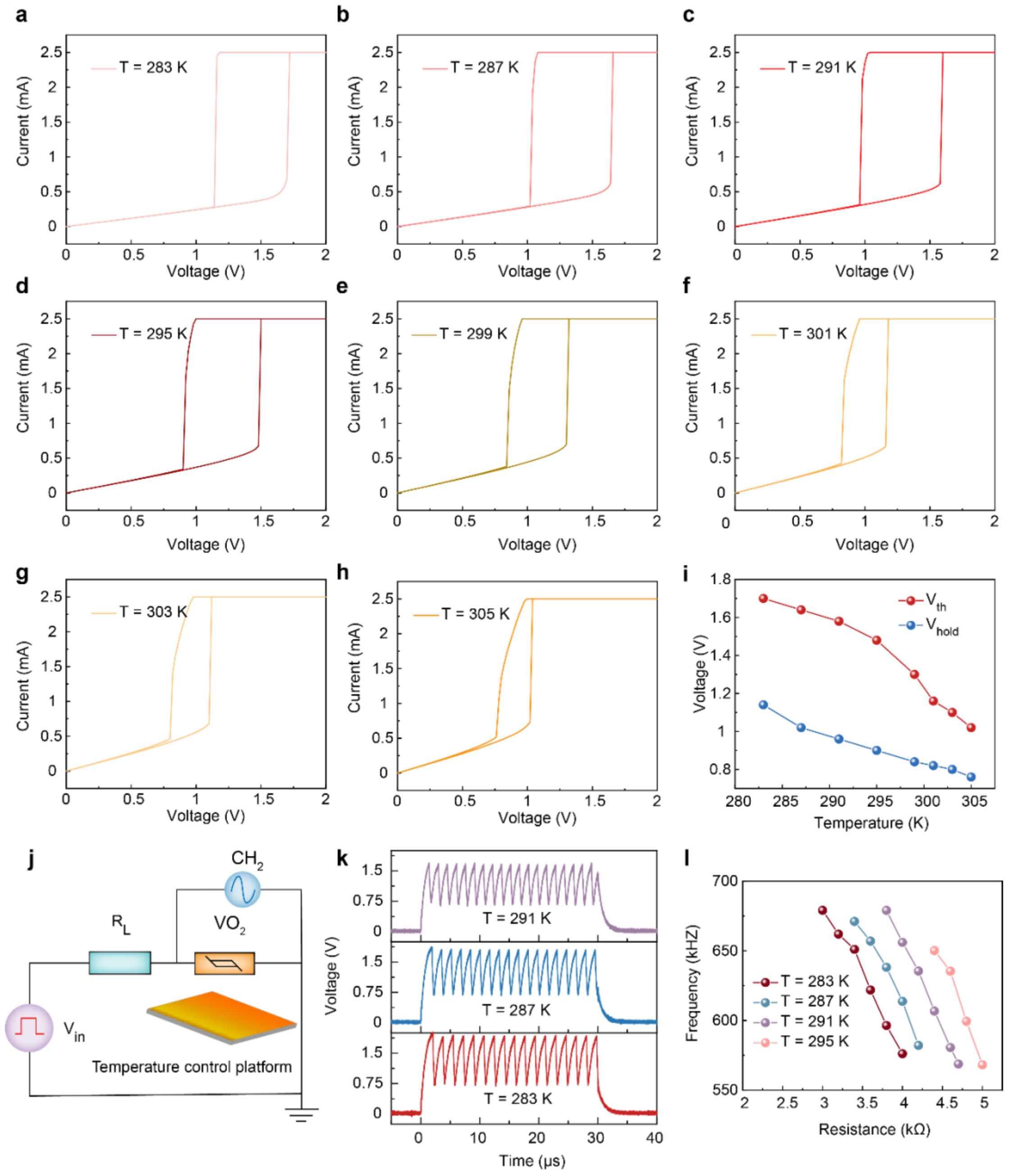

Supplementary Figure 22. The characteristics of the VO_2 memristor under different temperatures. (a)-(h) The *I*-*V* characteristics of devices with different temperature. The window of V_{th} - V_{hold} shrinks gradually with increasing temperature. **(i)** The V_{th} and V_{hold} at different temperatures. Both V_{th} and V_{hold} gradually decreases with increasing temperature. **(j)** Diagram of equipment to test firing frequency of spiking neuron at different temperatures. Devices are placed directly on a temperature-

controlled platform. **(k)** The firing frequency of neurons at different temperatures. With increasing temperature, the firing frequency gradually increased where input voltage is 5 V and R_L is 4 kΩ. **(l)** Spiking frequency as a function of load resistance (R_L) with different temperatures.

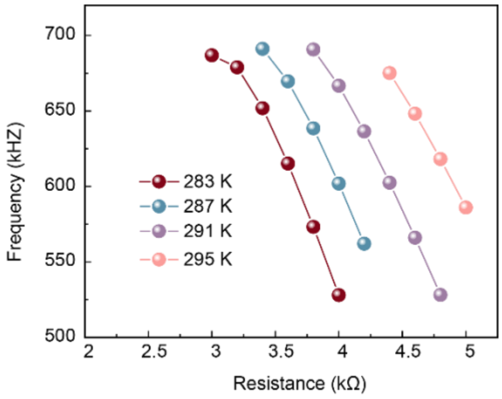

Supplementary Figure 23. Spiking frequency as a function of load resistance (R_L) with different temperatures calculated by the model.

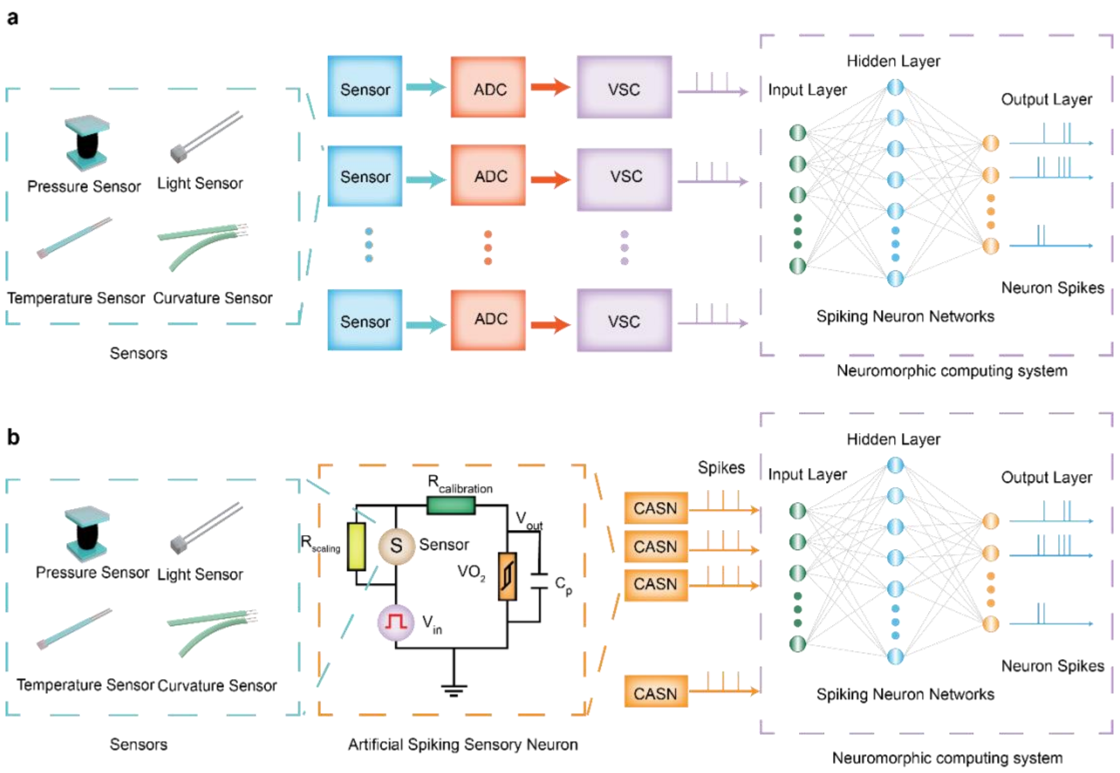

Supplementary Figure 24. Comparison between neuromorphic perception system based on silicon circuits and our system. (a) Schematic of the traditional neuromorphic perception system based on silicon circuits with lots of ADCs (analog-to-digital converters) and VSCs (voltage-to-spike converters). **(b)** Schematic of neuromorphic perception system based on our calibratable artificial sensory neurons.

Supplementary Table 1. Parameters used in the device COMSOL model.

Parameter	Value	Units
$\rho_{0,m}$	2.2×10^{-7}	$\Omega \cdot m$
$\rho_{0,r}$	1×10^{-6}	$\Omega \cdot m$
$E_{a,m}$	0.15	eV
$E_{a,r}$	0.067	eV
W	5	eV
A_h	2.6×10^{-77}	−
A_c	1.5×10^{-77}	−

Supplementary Table 2. The values of the parameters used in the modeling of spiking neuron considering the temperature.

Parameter	Value	Units
R_{off}	3100	Ω
R_{on}	450	Ω
R_{th}	31313	K/W
T_{t}	318.736	K
Vin	5	V
R_{L}	4	kΩ

Supplementary Table 3. Comparison of different spike-based sensory neurons.

Device		Epitaxial VO_2 (this work)	VO_2[1]	NbO_x[2]	NbO_x[3]	NbO_x[4]
Perception type	Pressure	√	√	√	×	√
	Light	√	×	×	√	×
	Temperature	√	×	×	×	×
	Curvature	√	×	×	×	×
Impedance matching with different sensors		√	×	×	×	×
Sensitivity		151.74 kHz/N, 0.13 kHz/Lux, 2.8 kHz/℃	60.8 kHz/kPa	/	/	/
SNR		33.66 dB (tactile), 31.90 dB (optical), 29.92 dB (temperature)	/	/	/	/
Energy consumption		2.9 nJ/spike	/	38 pJ/spike	/	~6 nJ/ spike
Highest spiking frequency		~1.3 MHz	174 kHz	~1.1 MHz	~2.2 MHz	~9 MHz

Supplementary Table 4. Circuit parameters in this article.

Figure No.	R_L	V_{in}	$C_{parallel}$	R_c	$R_{scaling}$
Fig. 3b,g	/	5 V	/	/	/
Fig .3c,h	4 kΩ	/	/	/	/
Fig. 3d,i	4 kΩ	5 V	/	/	/
Fig. 3k	/	5 V	/	0, 0.4 kΩ, 0.8 kΩ	/
Fig. 3l	/	5 V	/	0, 0.6 kΩ, 0, 0, 1.2 kΩ, 0	/
Fig. 4a-c	Pressure sensor	5 V	2000 pF	2.6 kΩ	4 kΩ
Fig. 4d-f	Light sensor	5 V	2000 pF	2.4 kΩ	3 kΩ
Fig. 4g-i	Temperature sensor	5 V	2000 pF	2.4 kΩ	4.5 kΩ
Fig. 5	Curvature sensor	5 V	2000 pF	0.4 kΩ, 0, 0, 0, 0.2 kΩ	5.8 kΩ

Supplementary Note 1: Simulation of VO_2 device in COMSOL

We simulated the operation of the VO_2 device in COMSOL Multiphysics software based on the metal-insulator transition (MIT) model described in ref. 5. In this model, the resistive switching process is simply due to the temperature change in the switching region resulting from the interplay of Joule-heating and the heat dissipation of the device. The thermally-activated high resistivity of VO_2 in the lower temperature range is given by Eq.1:

$$\rho_m = \rho_{0,m} \cdot \exp\left(\frac{E_{a,m}}{k_b T}\right) \tag{1}$$

where $E_{a,m}$ is the activation energy. For the low resistivity (ρ_r) region in the higher temperature range, we used the same equation with different values of $\rho_{0,r}$ and $E_{a,r}$. As both high and low resistivity phases coexist during the transition, the switching region can be regarded as a parallel circuit. Hence, the overall resistivity is given by Eq. 2:

$$\rho = \frac{\rho_r \rho_m}{f_r \rho_m + (1 - f_r)\rho_r} \tag{2}$$

where f_r is the volume fraction of the low resistivity phase and is given by Eq .3:

$$f_r = \frac{1}{1 + A \cdot \exp\left(\frac{W}{k_b T}\right)} \tag{3}$$

W is the energy scale of the MIT and is related to the steepness of the resistivity change. A is a constant related to the temperature at which the MIT takes place. The values of A during the heating process (A_h) and the cooling process (A_c) are different. The parameters in the equations above were tuned so that the simulated *I-V* curve fit the measured *I-V* curve of our device (Supplementary Table 1).

The materials used in the simulations were Al_2O_3 substrate and Au/Ti electrodes.

The dimensions of the switching region and the electrodes were also the same as our actual device. Other dimensions such as the thickness of the substrate as well as the length and width of the entire geometry were chosen to be large enough such that their effect on the MIT is negligible.

To investigate the effect of the series resistance on the spiking frequency, we simulated the artificial spiking neuron circuit using the model above. The resistor in series with the VO_2 device was varied from 3.4 kΩ to 5.2 kΩ in 200 Ω increments with a 10 μF parallel capacitor. After a sufficient number of spikes (10 spikes in our case), the frequency was extracted from the node voltage (voltage across the capacitor) waveform. Supplementary Fig. 12 shows the spiking frequency versus series resistance plot, illustrating an apparently linear relationship in the resistance range concerned. In addition, we simulated the response of the circuit to a 5V single pulse input signal. Supplementary Fig. 12 also shows the node voltages and the input signals of several circuits, each with a different series resistor. The ambient temperature in every simulation was kept constant at 300 K.

Supplementary Note 2: Structural and electrical characterization of epitaxial VO_2 devices

To characterize the epitaxial VO_2 memristor, the Microstructural and compositional characterization of the epitaxial VO_2 device is shown in Supplementary Fig. 1. Supplementary Fig. 1a shows the Cross-sectional STEM image of the device and the corresponding EDS mapping, where Al, O, Au, Ti, Si and V elements in the device and

their locations can be observed clearly. Supplementary Fig. 1b shows the EDS elemental line profile in the region of the device shown by the STEM image.

Supplementary Fig. 3 shows the transient switching response of the VO_2 threshold switching device. The switching speed of the VO_2 threshold switching device in this work is < 200 ns from off-state to on-state(Supplementary Fig. 3b) and < 75 ns from on-state to off-state(Supplementary Fig. 3c). Supplementary Fig. 4 shows the quantification of variability in positive and negative threshold/holding voltages in 1000 cycles. Supplementary Fig. 5a shows the I-V characteristics measured in 10 devices. The $V_{\mathrm{th\_pos}}$, $V_{\mathrm{hold\_pos}}$, $V_{\mathrm{th\_neg}}$, $V_{\mathrm{hold\_neg}}$ of 10 devices are shown in Supplementary Fig. 5b and the variability of $V_{\mathrm{th\_pos}}$, $V_{\mathrm{hold\_pos}}$, $V_{\mathrm{th\_neg}}$, $V_{\mathrm{hold\_neg}}$ in 10 epitaxial VO_2 devices are shown in Supplementary Fig. 5c-f.

Supplementary Note 3: Simulation of the spike-based neuromorphic perception computing system for pressure image recognition

A spike-based neuromorphic sensory computing system for pressure image recognition which consists of a 3-layer spiking neural network (SNN) and 784 artificial spiking tactile sensory neurons was built in Python based on experimental results using SpikingJelly[6]. The simulated SNN was composed of 784 input neurons, 196 hidden neurons, and 10 output neurons which corresponded to 10 possible classes (from 0 to 9). Epitaxial VO_2 devices are used as artificial spiking tactile sensory neurons (Fig. 4a) and LIF neurons of SNN where the parameters were extracted from experimental data (as shown in Supplementary Figure 20). The main parameters of the LIF neuron used

in the simulation are high resistance state (2.2 kΩ), parallel capacitor (5 μF), V_{th}(1.4 V), V_{hold}(0.85 V), the series resistance(4 kΩ), and state update time step (3.5 μs). To simulate the artificial spiking tactile sensory neurons, we fit the pressure and spiking frequency curve as shown in Supplementary Figure 20a.

Backpropagation (BP) was used to train the neural network online. Here, 784 spiking tactile sensory neurons are used to sense pressure and encode it into pulses of different frequencies. The value of each pixel in a handwritten digital image is regarded as pressure. First, we convert the pressure into spiking frequency through the fitted curve (Supplementary Fig. 20a). We can calculate the probability of spikes in each time step by comparing the spiking frequency with the length of the simulation time step. The higher the frequency, the greater the probability of spiking at each time step. During the simulation lasting for 50 time steps, there is a train of 50 spikes generated. Due to the existence of 784 pixels, a total of 784 spike trains will be generated with 50 steps. Next, 784 spike trains are sent to the linear layer of SNN step by step. At last, the output neurons spikes, and the index of the most frequently spiking neuron was taken as a prediction result. During the backpropagation process, the error is calculated by the label and the spiking rate of the last layer of neurons. As the activation function of the spiking neuron is a step function, which has an infinite gradient, a sigmoid-type surrogate function is used to calculate the gradient during the backpropagation process.

Supplementary Note 4: Correspondence of spiking frequency of artificial spiking neuron to the input voltage, series resistance R_L, and parallel capacitor $C_{parallal}$

In order to explore the influence of series resistance R_L on the spiking frequency, we fixed the applied voltage at 5 V and then changed the series resistance to observe how the frequency changes as shown in Supplementary Fig. 7. As the series resistance increases from 2.6 kΩ to 5 kΩ, the spiking frequency gradually decreases. This is because as the series resistance increases, the input current gradually decreases and the charge accumulation becomes slower. Supplementary Fig. 8 shows the output results of the spiking neuron under different applied voltages. As the applied voltage increases from 4.4 V to 7.2 V, the spiking frequency gradually increases. The output results of spiking neuron under different parallel capacitor shown in Supplementary Fig. 9. As the parallel capacitor increases, the firing frequency gradually decreases, this is because larger capacitance makes the integration process slower. Supplementary Fig. 10-11 shows the output results of the spiking neuron with different series resistance and applied voltage. A spiking frequency under 150 Hz is obtained which is similar to the human nervous system showing great potential in the field of human-machine interaction.

Supplementary Note 5: The spike-based neuromorphic sensory system for tactile, vision, and temperature perception.

We combine the spiking neuron with the pressure sensor, light sensor, and temperature sensor to form a spike-based neuromorphic perception system that can encode pressure, illuminance, and temperature into spikes. Supplementary Fig. 17 shows the spiking frequency response under different pressure. As the pressure

increases, the frequency of neuronal spiking increases, since as the pressure increases, the resistance of the pressure sensor decreases, increasing the input current, speeding up the charge accumulation. The spiking frequency response under different illuminance and temperatures are shown in Supplementary Fig. 18 and Supplementary Fig. 19 respectively. Greater illuminance and higher temperature both increase the frequency of spiking.

Supplementary Reference

1. Fang, S. L. *et al.* An Artificial Spiking Afferent Neuron System Achieved by 1M1S for Neuromorphic Computing. *IEEE Trans. Electron. Devices* 1-7 (2022).

2. Zhang, X. *et al.* An artificial spiking afferent nerve based on Mott memristors for neurorobotics. *Nat. Commun.* **11**, 51 (2020).

3. Wu, Q. *et al.* Spike Encoding with Optic Sensory Neurons Enable a Pulse Coupled Neural Network for Ultraviolet Image Segmentation. *Nano. Lett.* **20**, 8015-8023 (2020).

4. Li. F. *et al.* A Skin-Inspired Artificial Mechanoreceptor for Tactile Enhancement and Integration. *ACS Nano* **15**, 16422-16431 (2021).

5. Zhong, X., Zhang, X., Gupta, A. & LeClair, P. Avalanche breakdown in microscale VO_2 structures. *J. Appl. Phys.* **110**, 084516 (2011).

6. Fang, W. et al. SpikingJelly. https://github.com/fangwei123456/spikingjelly (2020).
